# Do we assess all relevant behaviours in children with cognitive impairments using the revised Face, Leg, Cry, Consolability (r-FLACC) Scale? – A Qualitative Study

**DOI:** 10.1007/s00431-025-06540-8

**Published:** 2025-10-20

**Authors:** Brigitte Messerer, Victoria Winkler, Marko Stijic, Alexander Avian

**Affiliations:** 1 Department of Anaesthesiology and Intensive Care Medicine, Medical University of Graz, Austria, Auenbruggerpl. 29, 8036 Graz, Austria; 2 Institute for Medical Informatics, Statistics and Documentation, Medical University of Gra, Auenbruggerpl. 2/5, 8036 Graz, Austria; 3 University Clinic for Neurology, Clinical Department for Neurogeriatrics, Medical University of Graz, Auenbruggerplatz 22, 8036 Graz, Austria

**Keywords:** R-FLACC, Intellectual disability, Development disability, Observational pain, Assessment children

## Abstract

For pain assessment in cognitive impaired children, the r-FLACC (revised-Face, Legs, Activity, Cry and Consolability) scale can be used simply and effectively postoperatively in clinical practice. The aim of this study was to analyse, whether all relevant behaviours for pain assessment in cognitive impaired children are covered by the r-FLACC. In this single-centre retrospective study, we analysed all r-FLACC tools from children/adolescents with cognitive impairment who underwent surgery at our department between February 2011 and December 2024. The objective was to find out what pain behaviours in children with cognitive impairment are not included in the r-FLACC questionnaire. The study included 531 observations from 345 patients (female: 45.5%; median age 9.8 years, age range 2.6–18 years). All specific behaviours within the r-FLACC categories were selected. A total of 1.514 pain-related behaviours were added to the existing r-FLACC categories. Of these behaviours, 444 were already mentioned in the r-FLACC as pain behaviours (207 in the same category and 237 in a higher or lower category). Of the remaining 1070 behaviours, 915 could be grouped into 10 categories that address extend the existing r-FLACC categories. The remaining 155 cases were put into four new categories: aggressive behaviour, physiological reaction, specific behaviour and posture. *Conclusions*: The current version of the r-FLACC covers most of the pain-related behaviours in cognitive impaired children/adolescents. Additional behaviours related to ‘Facial expression’, ‘Sound/communication’ and ‘Movement’ might be added to the existing categories. The behaviour of aggression, which has not yet been mentioned in the r-FLACC, could be included as a new dimension in a modified r-FLACC.

**Table:** 

**What is Known:** • *While some children with cognitive impairment are able to name their pain, others cannot and are unable to quantify the severity with words.* • *A suitable reliable instrument to assess pain in cognitive impairemt patients is the r-FLACC (revised- Face, Legs, Activity, Cry and Consolability).*
**What is New:** • *Ten new categories that extend the existing r-FLACC categories were found.* • *Three new categories could be identified: Aggressive behaviour, physiological reaction, posture.*

**What is Known:**

• *While some children with cognitive impairment are able to name their pain, others cannot and are unable to quantify the severity with words.*

• *A suitable reliable instrument to assess pain in cognitive impairemt patients is the r-FLACC (revised- Face, Legs, Activity, Cry and Consolability).*

**What is New:**

• *Ten new categories that extend the existing r-FLACC categories were found.*

• *Three new categories could be identified: Aggressive behaviour, physiological reaction, posture.*

## Introduction

The implementation of a routine pain assessment strategy is an essential component of effective pain management and a fundamental aspect of providing optimal care [1]. An excellent practical approach is the utilisation of pain scales, which facilitate a structured monitoring of the progress and success of pain therapy. We have to take into account the child’s age, their current cognitive capabilities and the simplicity of use in a clinical setting when selecting an optimal tool [2].

Verbal reporting is the ‘gold standard’ for pain assessment because pain is inherently subjective [3]. While some children with cognitive impairment are able to verbalise their pain, others lack this ability and are thus unable to report its severity [4]. In children with multiple disabilities, needs, wishes and interests are communicated non-verbally, in particular through certain behavioural expressions. Therefore, tools used to assess pain in cognitively impaired children should include a range of possible behavioural pain indicators [4, 5].

A suitable, reliable and valid instrument is the r-FLACC (revised-Face, Legs, Activity, Cry and Consolability) scale, which can be used simply and effectively postoperatively in clinical practice in children and adolescents with severe neurological deficits and severely limited communication [6]. The FLACC scale was originally developed as a simple behavioural assessment scale for young children between the ages of 2 months and 7 years who cannot or will not verbalize the intensity of their pain [7]. The tool contains five categories (Facial expression, Legs, Activity, Crying and Consolability) that are assumed to reflect the experience of pain in children [8]. Each category is scored from 0 to 2, resulting in a total score of 0 (no pain/relaxed/comfortable) to 10 (reflects the maximum level of pain/discomfort).

In 2006, Malviya et al. [9] revised the scale to better evaluate and manage postoperative pain in cognitive impaired children, as their behavioural reactions to a painful hurt may be very different from those of children with normal abilities. They incorporated several additional descriptors in the five categories that were most consistently associated with pain in cognitive impaired children/adolescents, aged up to 18 years. These included facial expression, verbal emotional outbursts, tremors and an increase in spasticity and altered breathing behaviour. Furthermore, the revised scale included an open descriptor under each category, which allows parents and caregivers to record unique, descriptive behaviours for each impaired child.

Gathering more information about the expression of pain in children with intellectual disabilities is of great importance and relevance, especially for the quality of care [10]. Those caring for these children are aware that inadequate pain management may be due to a lack of recognition [5]. Therefore, pain research in children with intellectual disabilities still focuses on pain assessment [11].

It seems possible that not all essential or crucial pain indicators for children with cognitive impairments are already included in the r-FLACC. Therefore, the aim of this study was to analyse, whether all relevant behaviours for pain assessment in cognitive impaired children are covered by the r-FLACC. To find answers, we retrospectively collected and all r-FLACC instruments from cognitively impaired children and adolescents treated in our Paediatric Surgery department over a period of almost fourteen years. All behaviours that were stated typically by parents for their children were analysed.

## Methods

Ethics approval was obtained from the University’s research ethics board (University Ethics committee of the Medical University of Graz, Austria, approved the study protocol (EC Nr. 33–602 ex 20/21).

### Participants

This retrospective study was conducted at the Department of Paediatric and Adolescent Surgery of the Medical University Hospital of Graz, Austria. We analysed all children with cognitive impairment who underwent surgery between February 2011 and December 2024 and whose pain was assessed using the German version of the revised-Face, Legs, Activity, Cry and Consolability (r-FLACC) scale ratings [12].

#### Study procedure

On admission, the nursing staff interviewed the parents, marked the representative pain behaviours in each category of the r-FLACC scale and recorded specific individual pain behaviours in the open descriptors under each category. The result was a patient-specific tool that gave all those involved in the patient's care a clear overview of the patient’s pain, from the initial assessment, through the examinations, surgical procedures, recovery room/intensive care unit, to discharge. After discharge from hospital, the r-FLACC was scanned and archived in our online data system. The scale was therefore already available for new admissions and only needed to be updated.

#### Material

In 2008, our department started a process to standardize pain assessment in children. In order to have a measurement instrument for cognitively impaired children whose metrics are comparable to those used for self- and peer-assessment of other children, the r-FLACC was selected as the most suitable for our department after testing different assessment methods in the interdisciplinary team. After a test phase, routine collection and systematic documentation began in February 2011. All r-FLACC documented from this point onwards were included in the analysis. According to an internal standard operating procedure, the pain nurses were required to collect all r-FLACC and deposit them in the ward secretary’s office upon discharge of the child.

We extracted the patient data (sex, date of birth) and the marked as well as the recorded individual behaviours in the five categories of the scale from the written r-FLACC forms into an Excel spreadsheet.

#### Analytical approach

Continuous data are reported as mean ± standard deviation or median and interquartile range (IQR). The age of each participant was calculated from the difference between the date of completion of each scale and the date of birth. For 77 participants, only the year of the interview was given, so 30 June was used as the basis for calculating age in years. Categorical data are presented as absolute and relative frequencies. Only patients with at least one response to the r-FLACC items were analysed. Missing data were not corrected.

First, we evaluated the number of original behavioural descriptors in the respective r-FLACC categories. In addition, the individual comments were analysed in detail. A qualitative content analysis was therefore carried out. The existing response categories and examples within these response categories were used for deductive coding. For comments that could not be categorized using this deductive approach, an inductive approach was chosen. These comments were screened and new categories were defined based on the frequency of similar comments. The inductive phase was conducted in an iterative manner, involving reading, discussion and re-reading. The definition of new categories was reached through a consensus among the authors. Finally, we thoroughly reviewed the reported behaviours to determine whether they were indeed inconsistent with the existing r-FLACC categories and whether they were in line with the new categories, we developed.

In order to avoid inaccuracies in the translation, the authors mutually agreed on the English wording of the behaviours given by the parents in German, which was then checked by a colleague whose native language is English. In order to avoid inaccuracies in the translation, the authors jointly agreed on the English wording of the behaviours given by the parents in German. Finally, a colleague who is a native English speaker checked the English wording.

Descriptive statistical analyses were performed with the Statistical Package for Social Sciences Version 22.0. (SPSS Inc., Chicago, IL, USA).

## Result

In this study, 345 patients (157 female; 45.5%) were included. Of our 345 patients, 88 were observed more than once, giving 531 observations. These patients underwent surgical or minimally invasive procedures in a hospital or outpatient setting. The maximum number of observations for one patient was 25. The median age at time of first observations was 9.8 years (range, 2.6–18.0 years; all observations: median 10.7, range 2.6–18.0) (Table 1).

**Table 1:** Sociodemographics for all patients at the first observation (*n* = 345) and all observations (*n* = 531)

		First observation of each child (*n* = 345)	All observations (*n* = 531)
Sex	female	157 (45.5%)	233 (43.9%)
	male	188 (54.5%)	298 (56.1%)
Age	≤ 6	92 (26.7%)	116 (21.8%)
	> 6–10	84 (24.3%)	130 (24.5%)
	> 10–14	98 (28.4%)	169 (31.8%)
	> 14–18	71 (20.6%)	116 (21.8%)

### Selected behaviours of the r-FLACC

Within the r-FLACC categories, all specified behaviours were selected (Table 2). Very frequently (> 40%), the following behaviours were chosen with a score of ‘0’ in the r-FLACC, LEGS: ‘normal position or relaxed’ (47.1%), CRY: ‘no cry’ (49.7%) and in CONSOLABILITY: ‘content’ (46.3%). Behaviours above 40% with a score of ‘1’ were in the category FACE: ‘occasional grimace’ (40.7%), CRY: ‘whimpers’ (49.0%), CONSOLABILITY: ‘reassured by occasional touching’ (45.8%) and with a score of ‘2’ in the category CRY: ‘crying steadily’ (49.3%) and screams (42.9%) and in the category CONSOLABILITY: ‘difficult to console’ (41.2%).

**Table 2:** Table 2 Frequency of the r-FLACC (revised-Face, Legs, Activity, Cry and Consolability) descriptors associated with pain (*n* = 494)

	Score 0		Score 1		Score 2	
	Behaviour	%	Behaviour	%	Behaviour	%
**Face**	No particular expression	38.4	Occasional grimace	40.7	Consistent grimace	25.4
	No smile	12.2	Occasional frown	21.3	Consistent frown	17.9
			Withdrawn	5.1	Frequent quivering chin/consistent quivering chin	7.3
			Disinterested	4.3	Clenched jaw	17.1
			Appears sad or appears worried	8.1	Distressed-looking face	9.4
					Expression of fright or expression of panic	5.6
**Legs**	Normal position or relaxed	47.1	Uneasy	33.7	Kicking	18.3
	Usual tone and motion to limbs	7.0	Restless	15.3	Legs drawn up	30.1
			Tense	32.8	Marked increase in spasticity	7.9
			Occasional tremors	2.6	Constant tremors	0.9
					Jerking	1.5
**Activity**	Lying quietly	26.9	Squirming	17.5	Arched	21.7
	Normal position	27.5	Shifting back and forth	9.6	Rigid	11.9
	Move easily	17.6	Tense movements	5.5	Jerking	13.6
	Regular, rhythmic respirations	5.1	Guarded movements	2.4	Severe agitation	1.9
			Intermittent sighs	5.6	Head banging	3.6
			Head back and forth	5.1	Shivering (not rigors)	4.1
			Shallow or splinting respirations	3.1	Breath holding	1.9
			Mildly agitated	0.8	Gasping or sharp intake of breaths	2.8
					Sever splinting	1.1
**Cry**	No cry	49.7	Moans or	34.7	Crying steadily	49.3
	No verbalization	16.8	Whimpers	49.0	Screams	42.9
			Occasional complaint	15.3	Sobs	11.5
			occasional verbal outburst	1.9	Frequent complaints	11.5
			grunt	1.3	Repeated outburst	3.8
					Constant grunting	6.2
**Consolability**	Content	46.3	Reassured by occasional touching	45.8	Difficult to console	41.2
	Relaxed	37.7	Hugging	29.4	Difficult to comfort	29.6
			Being talked to	32.4	Pushing away caregivers	6.2
			Distractible	19.4	Resisting care	2.6
					Resisting comfort measures	4.0

### Additional reported behaviour patterns that are partially covered by the r-FLACC

In total, 1.514 pain-related behaviours were added to the existing r-FLACC items for the 345 patients (293 behaviours for r-FLACC score 0, 475 for score 1 and 746 for score 2). Of these stated behaviours, 444 could be classified as a pain behaviour already mentioned in the r-FLACC (207 within the same pain category and 237 in a higher or lower pain category).

**Fig. 1:**
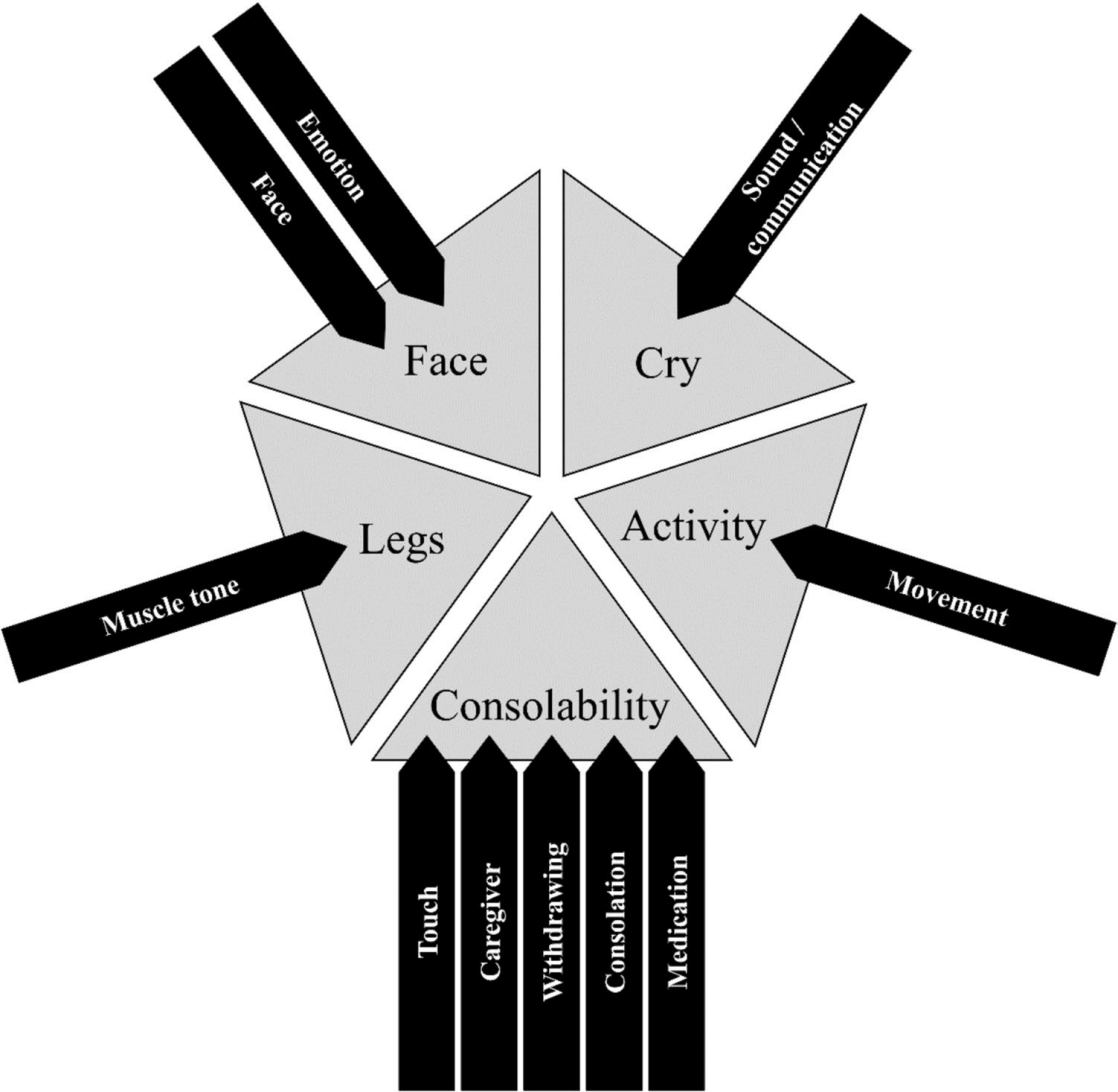
Original dimensions of the r-FLACC (gray) and new categories (black)

Of the remaining 1.070 individually reported behaviours (Table 3), 915 could be grouped into 10 categories (Fig. 1) that address topics partially covered by r-FLACC and thus extend the existing r-FLACC categories: The categories ‘*Face*’ (*n* = 217) and ‘*Emotion*’ (*n* = 19) represent extensions of the r-FLACC dimension FACE. These two categories add the more detailed descriptions of the face (e.g. changes in the oral area or slight to strong grimacing) and other emotions (e.g. joy in the absence of pain and anger in pain). The category ‘*Muscle tone*’ (*n* = 93) encompasses more than just the r-FLACC dimension LEGS. It also includes the general appearance of the entire body in the absence and presence of pain. The category ‘Movement’ (*n* = 179) contributes to the r-FLACC dimension ACTIVITY. This includes jerky movements and fidgeting, as well as specific movements such as hands that are constantly active, or movements of the hands but not of the legs. The category ‘Sound/communication’ (*n* = 204) also includes non-verbal communication, such as pointing to a painful area. In addition, vocalization, chatting and communicative mood in the absence of pain are aspects not covered by the r-FLACC dimension CRY.

**Table 3:** Individual behaviours not listed in r-FLACC (*n* = 915), grouped into 10 categories that address issues partially covered by r-FLACC, extending the existing r-FLACC categories

r-FLACC categories	**New created category**		**Score 0**	**Score 1**	**Score 2**
**Face**	Face (*n* = 217)	*n*	*n* = 128	*n* = 36	*n* = 53
		Examples	• Mouth relaxed • Smiles	• Slight grimacing • Teeth grinding	• Trembling lips • Strong grimacing
	Emotion (*n* = 19)	*n*	*n* = 8	*n* = 4	*n* = 7
		Examples	• Satisfied facial expression • Cheerful	• Seems angry	• Is annoyed • Anger
**Legs**	Muscle tone (*n* = 93)	*n*	*n* = 12	*n* = 19	*n* = 62
		Examples	• Goes into a stretched state when excited or CURIOUS • Relaxed leg position	• Hyperextends the legs • Stretches extremely through	• Let everything hang down • Stretching into a hollow back
**Activity**	Movement (*n* = 179)	*n*	*n* = 32	*n* = 68	*n* = 79
		Examples	• Legs stay the way you put them down • Hands constantly active	• Rather jerky movements • Fidget	• Unsteady rocking • Moves the hands but not the legs
**Cry**	Sound/communication (*n* = 204)	*n*	*n* = 33	*n* = 82	*n* = 89
		Examples	• Makes sounds • Chats, is communicative	• Points to painful areas • Scratches areas that hurt • roars	• Tears • Cries with voice • Speaks no more
**CONSOLABILITY**	Touch (*n* = 85)	*n*	*n* = 4	*n* = 47	*n* = 34
		Examples	• Stroking the face • Touching the hand • Seeks physical contact	• Mom talks and strokes • Touching the arm	• Seeks physical contact • Lift up
	Caregiver (*n* = 72)	*n*	*n* = 1	*n* = 30	*n* = 41
		Examples	• can be well soothed by the mother	• Seeks comfort and closeness with a caregiver • Does not calm down with strangers	• Mother can reassure • Needs parents
	Withdrawing (*n* = 20)	*n*	*n* = 2	*n* = 9	*n* = 9
		Examples	• pulls the blanket over the head	• Turns away • Avoids eye contact • Wants to sleep • Wants to be left alone	• Pulls legs away • Very withdrawn • Only wants peace and quiet, says the mother
	Consolation (*n* = 21)	*n*	*n* = 1	*n* = 8	*n* = 12
		Examples	• easy to calm	• Presses fist against cheek • Pushes the head into the pillow	• Can not be calmed • Let crawl
	Medication (*n* = 5)	*n*	*n* = 0	*n* = 1	*n* = 4
		Examples		• Medication makes him/her calmer	• With painkillers • Medication required

The categories ‘*Touch*’ (*n* = 85), ‘*Caregiver*’ (*n* = 72), ‘*Withdrawing*’ (*n* = 20), ‘*Consolation*’ (*n* = 21) and ‘*Medication*’ (*n* = 5; primarily focused on pain medication), add to the r-FLACC dimension CONSOLABILITY aspects such as the type of physical contact (e.g. stroking the face, touching the hand or arm, seeking physical contact, lifting up), the importance of the parent/caregiver, preference for being alone (withdrawal), ways of comforting (consolation) and the fact that sometimes only painkillers can help.

### Additionally reported behaviour patterns that are not covered by the r-FLACC

Categories that are not covered by the r-FLACC and cannot be classified as an extension of existing categories (*n* = 155) were grouped in four categories: ‘*Aggressive behaviour*’ (*n* = 62), ‘*Physiological reaction*’ (*n* = 32), ‘*Specific behaviour*’ (*n* = 50) and ‘*Posture*’ (*n* = 11) (Table 4). The category ‘*Aggressive behaviour*’ includes both self-aggressive behaviour and aggressive behaviour directed at others. The main ‘*Physiological reactions*’ reported were sweating, changes in breathing and changes in skin colour. The aspects summarised in the categories ‘*Specific behaviour*’ and ‘*Posture*’ encompassed a diverse range of individual behaviours and body postures.

**Table 4:** Individual behaviours not covered in r-FLACC (*n* = 155), grouped into four new categories

Additionally reported behaviour patterns (*n* = 155) that are not covered by the r-FLACC				
Aggressive behaviour (*n* = 62)	*n*	*n* = 4	*n* = 15	*n* = 43
	examples	• Bites left thumb	• Throws things • Beats with the hands	• Bites into the lips • Hits himself/herself on the head
Physiological reaction (*n* = 32)	*n*	*n* = 2	*n* = 7	*n* = 23
	examples	• Breathes regularly • Flushed cheeks	• Breathing pauses • Sweating	• Breathing and pulse accelerated • Turns blue
Specific behaviour (*n* = 50)	*n*	*n* = 19	*n* = 12	*n* = 19
	examples	• Looks around the room • Mother interprets his/her own language	• Limps when walking • Yawns with the left hand over the mouth	• Hands to head • Gripes the ears
Posture (*n* = 11)	*n*	*n* = 7	*n* = 3	*n* = 1
	examples	• When spoken to, he/she straightens up • Sits in bed	• Lies in prone position • Lies sideways	• Lies on the stomach

## Discussion

In the present study, all of the descriptions of pain offered in the r-FLACC were selected. The most frequently observed (> 40%) behaviours associated with pain were ‘whimpers’, ‘uneasy legs’, ‘crying steadily’, ‘reassured by occasional touching’, ‘screams’, ‘difficult to console’ and ‘occasional grimace’. The behaviours in the open field could be grouped into 10 categories that address issues not covered by r-FLACC. They are named as the ‘Face’, ‘Emotion’, changes in ‘Muscle tone’, ‘Movement’, ‘Sound/communication’, ‘Touch’, reaction to ‘Caregiver’, ‘Withdrawing’, ‘Consolation’ and the need of ‘Medication’.

### r-FLACC scale behaviours that occur least frequently

Behaviours from the categories LEGS (regarding tremor and jerking) and ACTIVITY (regarding ‘mildly agitated’, ‘guarded movements’, ‘shallow or splinting respirations’, ‘severe agitation’, breathing abnormalities and ‘head banging’) were the least frequently reported (< 5%) pain indicators. The low level of agreement between LEGS and ACTIVITY is also in line with the findings of Voepel-Lewis et al. [13] and is likely related to the physical limitations that are common in this population of children. Malviya et al. [9] also pointed out that the lower agreement in these subcategories of existing pain instruments may be partly explained by the presence of underlying motor impairments, including spasticity, which may confound behavioural observations.

Other rarely selected behaviours were in the category FACE ‘disinterested’, in CONSOLABILITY ‘resisting care’ and in CRY ‘occasional verbal outburst’, ‘repeated outburst’ and ‘grunt’. The question thus arises as to whether these behavioural descriptions should remain anchored in the scale. However, for children who are not able to communicate the intensity of the pain verbally, behavioural cues remain the most reliable indicators of pain [14, 15].

The difficulty with observational pain assessment is that there may be a difference between what clinicians and parents expect and what they actually observe, as healthcare professionals usually encounter their patients in an extreme situation (e.g. during surgery, in the recovery room or in intensive care). In addition, crying or body movements can be caused by anxiety and discomfort, not just pain in patients who are unable to communicate [7].

### Individualized reported behaviour indicators that are partially covered by the r-FLACC

All participants added unique pain characteristics in all five r-FLACC categories, which we grouped into 10 new categories. The categories of ‘*Face*’, ‘*Sound/Communication*’ and ‘Movement’, which overlap strongly with the existing r-FLACC categories FACE, CRY and ACTIVITY respectively, were assigned the largest number of behaviours.

#### Face

The FLACC scale was evaluated in children with cognitive impairment, showing strong correlations in the ‘FACE’ and ‘CRY’ categories [13] and the ‘FACE’ category demonstrated the highest inter-rate reliability in the r-FLACC. Facial expression may not be reliable in children with cognitive impairment who are unable to verbalise and/or have physical limitations due to muscle tone, nerve damage or paradoxical pain behaviours [3]. A study by Messmer et al. [16] observed children with autism during venepunctures. Facial expression had the greatest effect on pain intensity ratings, regardless of information about pain sensitivity. These findings indicate a tendency to rely on facial expression as a primary pain indicator.

#### Sound/communication

In contrast to facial expressions, there is limited evidence on the association between vocalisation, which is a multifactorial phenomenon, and pain. However, Helmer et al. [17] indicated that vocalisation alone is only a limited indicator, and is more reliable with other pain indicators. They also pointed out that there is little evidence to distinguish which vocalisations are less or more associated with pain.

Children with profound intellectual and multiple disabilities more often make penetrating sounds of restlessness and it can be questioned whether these behaviours are truly painful or, rather more uncomfortable or embarrassing [18].

#### Movement

Movement is a feature of many pain assessment tools for children with cognitive impairment [9, 19, 20]. The newly created category ‘Movement’ has a large overlap with the ACTIVITY category of the r-FLACC and includes jerky movements and fidgeting as well as specific movements such as hands that are constantly active or movements of the hands but not the legs.

#### Muscle tone

The category ‘*Muscle tone*’ played a minor role in our study, although it has been an important aspect of pain assessment in several studies [21]. Some aspects of muscle tone are already included in the r-FLACC. The characteristics of altered muscle tone can be found in the category FACE (constant quivering chin and clenched jaw), in the category ACTIVITY (tense movements, rigid, jerking) and in the category LEG (normal position, tense, occasional tremors, marked increase in spasticity, constant tremor, jerking) [9]. However, the r-FLACC takes little account of the muscle tone of the rest of the body, particularly the general appearance of the whole body in the absence and presence of pain. Solodiuk [21] examined the pain responses reported by parents of children with intellectual disabilities and was able to identify several different categories of pain responses, including muscle tone, which showed an opposite response to pain along with vocalisation, social behaviour and activity level. Hauer and Solodiuk [22] found that 86% of the children with central nervous system impairments had increased muscle tone due to recurrent pain episodes. Altered muscle tone was found in the form of tension, back pain and stiffness of the extremities, suggesting that pain may act as a trigger for increased muscle tone in this population. Fox et al. [23] noted that the r-FLACC may not be suitable for people with cerebral palsy as some behaviours, such as ‘legs drawn up’ and ‘arched’ are common positions observed in people with spastic cerebral palsy, in the absence of pain. Besides, Wong found no relationship between pain and spastic, indicating that the pain modulating mechanism is mediated through either musculoskeletal overload or the inhibition of the release of the neurotransmitters in pain. Sighs of pain can be discrete and subject-specific, thus overlooked [24].

#### Withdawn behaviour

Parents/caregivers reported further descriptors that related to social withdrawal (e.g. ‘wants peace and quiet’, turns away’, ‘wants to be left alone’). The r-FLACC scale includes social withdrawal aspects in the FACE category (‘withdrawn’, ‘disinterested’) and in the CONSOLABILITY category (‘pushing away caregiver’, ‘resisting care or comforting measures’). During the revision of the FLACC by Malviya et al. [9], parents indicated ‘distant and unresponsive when in pain’ was an individual behaviour.

### Additionally reported behaviour patterns that are not covered by the r-FLACC

In the open-ended fields, there were 155 pain indicators that could not assigned to one of the defined categories. This is further evidence that pain reactions are very individual. Ranger and Campbell-Yeo described pain as an individual, subjective and lived experience [25]. Fillingim [26] also noted the considerable inter-individual variability in pain responses, citing demographic, genetic and psychosocial factors as key contributing elements. We defined four distinct categories: ‘*Aggressive behaviour*’, ‘*Physiological reactions*’, ‘*Specific behaviour*’ and ‘*Posture*’.

#### Aggressive behaviour

The aggressive behaviours reported were both auto aggressive (e.g. ‘beating the lip’, ‘hits oneself on the head’) and directed at others (e.g. ‘throw things’, beats with the hands’, ‘pinches others’, striking with the legs’).

Some aspects of aggression are already included in the r- FLACC, such as ‘kicking’ in the LEGS category and ‘aggression’ and ‘head-banging’ in the ACTIVITY category [9]. However, many aspects of aggressive behaviour are missing, especially in relation to auto-aggression. These could be important aspects of pain assessment in non-verbal children.

Aggressive behaviour is often associated with the experience of pain, according to studies examining the relationship between pain and aggressive behaviour in different populations and situations. Al-Khotani et al. [27] used the Arabic version of the Child Behaviour Checklist to examine the association between emotional and behavioural aspects in children and adolescents (10–18 years) with different types of temporomandibular disorders. They found that children who are in pain due to their disorder diagnosis show externalising problems such as aggressive behaviour. Wilde et al. [28] conducted a complex investigation of self-injurious behaviour in patients with tuberous sclerosis complex and found that these patients are at risk of developing self-injurious behaviour and, once developed, this behaviour is likely to be persistent. Another study by Eden et al. [29] found that 27% of children with tuberous sclerosis exhibit self-injurious behaviour and 50% are aggressive.

In autistic and intellectually disabled children, Courtemanche et al. [30] found that children with self-injurious and aggressive behaviours had significantly higher pain scores than those without self-injurious behaviour. Carr and Owen-Deschryver [31] found that problem behaviours such as self-injury, aggression, property destruction and/or tantrum were more common on sick days than on days when patients with developmental disabilities (4–21 years) were well. This was explained by higher levels of pain and discomfort on sick days.

Taken together, these findings suggest that aggressive behaviour is an important aspect of pain. This should be considered when assessing pain in different patient groups, particularly when assessing pain in non-verbal children.

### Implications

The r-FLACC in its current form already covers a very wide range of relevant behaviours. Nevertheless, behaviours were found that, on the one hand, expand the existing dimensions and, on the other hand, cannot be assigned to any existing ones. Therefore, from our point of view, a sensible solution would be to include those ten aspects that overlap with the existing dimensions and to name them as further example behaviour. This is easily possible for CRY, ACTIVITY and CONSOLABILITY. The dimension FACE could be renamed Face/Emotion, since there has also been a relevant expansion of the content here. Likewise, the new behaviours in the area of muscle tone do not only refer to the leg area, which is why a renaming to legs/muscle tone is suggested here. Of the four additionally reported behaviour patterns that are not covered by the r-FLACC, three patterns are very specific and were mentioned less frequently than 10% of the time. In contrast, aggressive behaviour was mentioned more frequently. Therefore, in our view, aggressive behaviour should be included as a new category. How to handle the changed metric from 0–12 to the previous 0–10 still needs to be discussed. Perhaps this area could be included in the ACTIVITY dimension and prominently highlighted here with example behaviours.

## Limitations

The study was conducted in a single hospital, which may limit generalisability. Not collecting information on the type and severity of cognitive impairment is another potential limitation of our study. In children with autism spectrum disorders for example, there is great heterogeneity in the expression (or lack of expression) of pain: patients may exhibit hyper- and hyposensitivity to pain, to no obvious signs of pain [32]. They show some qualitative impairments in language skills and express their pain through stereotypical behaviour that can be misinterpreted. Future research is needed in this area. In addition, there is still a lack of description of pain in children with cerebral palsy [33].

A limitation may be the lack of racial/ethnic diversity and possible implications for children/adolescents with cognitive impairment (i.e. compounded risk for inequalities), suggesting areas for future research improvement [34].

A further limitation is that it is possible that not all r-FLACC from the period 2011–2024 were included in the analysis, as some may be missing. The number of missing ones is likely to be very small, since there was a standardized procedure for collecting the r-FLACC. Furthermore, the influence of possible missing r-FLACC on the results is likely to be small, since it can be assumed that it is missing at random and the number of included r-FLACC is very high.

## Conclusions

In summary, the current version of the r-FLACC covers a large proportion of pain-related behaviours in cognitively impaired children/adolescents. As some of the behaviours covered (‘disinterest’, ‘resisting care’, ‘occasional verbal/repetitive outbursts’, ‘grunt’) are rarely reported, the question arises as to whether these behavioural descriptions should remain anchored in the scale. Consideration may be given to whether additional behaviours related to ‘*Facial expression*’, ‘Sound/communication’ and ‘Movement’ should be added to the existing categories.

All these findings emphasise the importance of having an observational pain assessment tool for each child with nonverbal pain, in which specific standardized, as well as unique, descriptive behaviours can be noted by parents/caregivers who know the child/adolescent well.

## Data Availability

The data that support the findings of this study are not openly available due to reasons of sensitivity and are available from the corresponding author upon reasonable request. Data are located in controlled access data storage at Meedical University of Graz.

## Abbreviations

IQR: Interquartile range
r-FLACC: Revised-Face, Legs, Activity, Cry and Consolability
